# Use of multiple metrics and clustering analysis to assess antimicrobial use in Shanxi hospitals, China: a cross-sectional study based on 25 general hospitals

**DOI:** 10.3389/fpubh.2025.1464613

**Published:** 2025-08-13

**Authors:** Donghong Yin, Yang Tang, Song Wang, Shuyun Wang, Ruigang Hou, Jinju Duan

**Affiliations:** ^1^Department of Pharmacy, Second Hospital of Shanxi Medical University, Taiyuan, Shanxi, China; ^2^Shanxi Pharmacy Administration & Quality Control Center, Taiyuan, Shanxi, China; ^3^Department of Pharmacy, The Affiliated Tianfu Hospital of Southwest Medical University, Meishan, Sichuan, China; ^4^Department of Pharmacy, School of Shanxi Medical University, Taiyuan, Shanxi, China

**Keywords:** antimicrobial use, public hospitals, antimicrobial stewardship, clustering analysis, retrospective study

## Abstract

**Objective:**

To investigate the current patterns of antimicrobial use among nonsurgical inpatients across 25 general hospitals in Shanxi Province and to evaluate the antimicrobial use rate, antimicrobial use density (AUD), days of therapy (DOT), length of therapy (LOT), and the application of cluster analysis in monitoring antimicrobial prescribing practices.

**Methods:**

This study included 25 general hospitals covering 11 cities in Shanxi Province. In total, 2064 hospitalized nonsurgical patients were evaluated for antimicrobial use between December 1, 2022, and January 31, 2023. Data collected included the proportion of antimicrobial prescriptions, antimicrobial use rate, AUD, DOT, and LOT. Statistical analyses were conducted using IBM SPSS version 21.0. Cluster analysis was employed to categorize the 25 hospitals systematically.

**Results:**

Among the hospitals, the antimicrobial utilization rate ranged from 43.00 to 83.33%. The intensity of antimicrobial use ranged from 40DDDs/ 100pd to 98.99DDDs/100pd. DOT values ranged from 380/1000pd to 713/1000pd, while LOT ranged from 425/1000pd to 1,014/1000pd. The top three antimicrobial classes by AUD were third-generation cephalosporins (15.38 DDDs/100pd), quinolones (13.60 DDDs/100pd), and cephalosporins (11.54 DDDs/100pd). The ICU had the highest antimicrobial use rate and AUD—91.67% and 133.28 DDDs/100pd, respectively —and the longest DOT (1,230/1000 pd). The infection department recorded the highest LOT (988/1000pd). In pediatrics, the AUD and DOT were 53.77DDDs/ 100pd and 1,106/1000pd, respectively. The 25 hospitals were grouped into three distinct clusters via cluster analysis. Statistically significant differences in some antimicrobial indicators were observed among the groups (*p* < 0.05).

**Conclusion:**

Across the 25 hospitals, the rate and intensity of antimicrobial use were relatively high in institutions and departments. During the study period, the use of cefoperazone/sulbactam and fluoroquinolones increased. Concurrently, the combined use of AUD and DOT provided complementary perspectives for evaluating antimicrobial consumption, allowing for a more comprehensive understanding of exposure levels across hospitals and departments. Cluster analysis provides valuable insights for identifying patterns into antimicrobial management and usage.

## 1 Introduction

The global rise of antimicrobial-resistant bacteria is accelerating due to the widespread use of antimicrobial drugs, and China is no exception (1). The overall drug resistance rate remains high, posing a significant threat to effective clinical treatment. Current estimates indicate that causes at least 700,000 deaths annually worldwide, a figure projected to reach 10 million per year by 2025, thereby representing a severe risk to public health and human safety (2). Inappropriate antimicrobial use has emerged as a critical global public health concern that must be urgently reduced. Without effective measures to curb antimicrobial misuse and suppress the spread of resistant pathogens, patient outcomes will continue to deteriorate, resulting in prolonged hospitalizations, higher mortality rates, and increased healthcare costs (3–5).

The Chinese government has consistently prioritized the rational use of antimicrobials and has strengthened drug management through policy guidance, technical support, data surveillance, supervision and inspection, as well as public education (6–9). To support clinicians in improving infectious disease diagnosis and treatment while preventing antimicrobial misuse, the Guiding Principles for Clinical Application of Antimicrobial and the National Guidelines for Antimicrobial Therapy have been successively issued and regularly updated (6, 10). Efforts have also been made to collect, sort, and analyze national antimicrobial surveillance data (11). Key antimicrobial indicators, such as the consumption (defined daily doses, DDDs) and usage intensity (i.e., DDDs/100 patient-days; pd), are statistically assessed across medical institutions, provincial units, and nationwide to determine whether antimicrobial use remains within established surveillance thresholds. In alignment with the goal of the World Health Organization to increase public awareness of antimicrobial resistance, an “Antimicrobial Awareness Week” campaign has been implemented (12). In 2022, 13 ministries and commissions, including the National Health Commission, Ministry of Education, and Ministry of Science and Technology, jointly issued the National Action Plan for the Containment of Microbial Resistance (2022–2025). This plan emphasizes improving the monitoring and evaluation system to support enhanced rational use of antimicrobials and to strengthen scientific and technological research on the prevention and control of microbial resistance (13, 14).

Since the outbreak of various respiratory pathogens in 2022, clinical scenarios have become more complex, leading to increased antimicrobial use. While managing respiratory pathogen epidemics involves multiple competing priorities, rational management of antimicrobial remains limited. Therefore, this multicenter cross-sectional study aims to calculate antimicrobial monitoring indicators, including DDDs and days of therapy (DOT), across hospitals and departments. We also analyzed the use of various antimicrobial agents to obtain accurate information regarding antimicrobial usage patterns. The findings could provide insights into developing and implementing tailored strategies and interventions at provincial and national levels to promote rational antimicrobial use during future health emergencies.

## 2 Materials and methods

### 2.1 Study design and setting

This study employed a cross-sectional design combined with real-world collection through a case-based investigation. The lead research hospital developed the ‘Special Questionnaire on Antimicrobial Use and Intravenous Infusion in Nonsurgical Patients in Shanxi Province. Each participating branch center designated researchers and case reporters to complete the paper-based questionnaire using standardized methods. The leading research hospital appointed data checkers to electronically review the submitted questionnaires, input the data into an Excel database, and conduct data verification, quality control, statistical analysis, and summarization.

This multicenter, retrospective, cross-sectional study on antimicrobial use was conducted across 25 hospitals in Shanxi Province between December 1, 2022, and January 31, 2023. It included 3 provincial-level tertiary hospitals, 11 prefecture-level tertiary hospitals, and 11 prefecture-level secondary hospitals. All cases during the study period were selected from the electronic medical record system, before being screened using random sampling. In total, 100 survey forms were completed in tertiary hospitals and 50 in secondary hospitals, and data on the demographic characteristics of patients and their antimicrobial use were collected. The following antimicrobial management indicators were statistically analyzed to monitor antimicrobial use and guide the development and improvement of rational use strategies: utilization rate, antimicrobial use intensity (AUI), DOT, and length of therapy (LOT).

### 2.2 Inclusion and exclusion criteria

Data analysis was performed on medication orders (including those not involving antimicrobials) and patient demographic and clinical information obtained from the electronic medical record system. The sample included patients randomly selected from 25 hospitals during the study period. Standard inclusion and sampling criteria were as follows: nonsurgical patients discharged between December 1, 2022, and January 31, 2023, after a hospital stay of ≥3 days. Surgical and interventional procedures were defined according to the National Clinical Version 3.0 Surgical Operation Classification Code (2022 summary version).

### 2.3 Outcome measurement

Primary outcomes included the distribution of antimicrobial use, reported individually or by category, as well as antimicrobial use indicators: DOT, LOT (1,000 pd), and the DOT/LOT ratio. When only one class of antimicrobial was used in a hospital, the specific drug name was reported instead of the class. Secondary outcomes included the percentage of patients using an antimicrobial, AUI, the proportion of antimicrobial prescriptions, the proportion of intravenous (IV) versus oral prescriptions, and the proportion of antimicrobial drug categories (restricted, unrestricted, and special use).

### 2.4 Statistical analysis

IBM SPSS Statistics version 21.0 was used for data analysis. Cluster analysis was performed to systematically group the 25 hospitals. ANOVA and the Kruskal–Wallis H test were used to assess intergroup differences. A *p*-value of < 0.05 was considered statistically significant. The Mann–Whitney U test with Bonferroni correction was used for *post-hoc* comparisons.

### 2.5 Ethical review

This study was reviewed and approved by the ethics committees of all 25 participating hospitals, including the Second Hospital of Shanxi Medical University (Center Ethics Approval reference number [2023] YX-334). Personally identifiable information—including patient names, ID numbers, and hospitalization numbers—was not recorded (only coded identifiers were used). All other personal data were kept strictly confidential. Throughout the data collection, the hospitals were numbered, and their names were anonymized. Since the study did not involve changes to treatment regimens for patients, 23 hospitals were granted ethical exemptions. Two hospitals required verbal informed consent, which was obtained by telephone calls. During the call, the patients were informed of the purpose of the study and reminded of their right to decline participation.

## 3 Results

### 3.1 Overall use of antimicrobial agents in 25 hospitals

In total, 2,064 nonsurgical inpatients with 18,187 medication orders were included in this study. Among these patients, 1,176 (56.98%) were administered one or more antimicrobial agents during hospitalization. Antimicrobial prescriptions accounted for 10.66% (1,939/18,187) of all medication orders. Based on prescription frequency, the five most commonly used antimicrobial classes were as follows: third-generation cephalosporins at 25.48% (494/1,939), quinolones at 23.21% (450/1,939), cefoperazone and sulbactam at 12.58% (244/1,939), penicillins at 10.62% (206/1,939), and combinations of penicillins, including *β*-lactamase inhibitors at 8.77% (170/1,939). Among the 1,164 individual antimicrobial prescriptions, 95.62% (1,113/1,164) were administered via IV infusion, while 4.38% (51/1,164) were administered orally.

The patients were administered 508 days of antimicrobial treatment per 1,000 days (LOT = 508/1,000pd), and DOT was 621/1,000pd. Among all antimicrobial agents, third-generation cephalosporins had the longest duration of use at 160/1,000pd, followed by quinolones (143/1,000pd), cefoperazone and sulbactam (83/1,000pd), penicillins (63/1,000pd), and combinations of penicillins, including *β*-lactamase inhibitors (56/1,000pd). The overall DOT/LOT ratio was 1.22, indicating that patients were administered an average of 1.22 antimicrobial agents during hospitalization.

Among all provinces, the cumulative DDDs of antimicrobials were 12,154.61, with 20,069 total hospital days, resulting in an overall AUI of 60.56 DDDs/100pd. The five most frequently used antimicrobial classes were third-generation cephalosporins (15.38 DDDs/100pd), quinolones (13.60 DDDs/100pd), cephalosporins (11.54 DDDs/100pd), penicillins (5.45 DDDs/100pd), and combinations of penicillins, including *β*-lactamase inhibitors (4.29 DDDs/100pd). The AUI for carbapenems was 1.29 DDDs/100pd.

### 3.2 Use of antimicrobials in different hospitals

**Figure 1:**
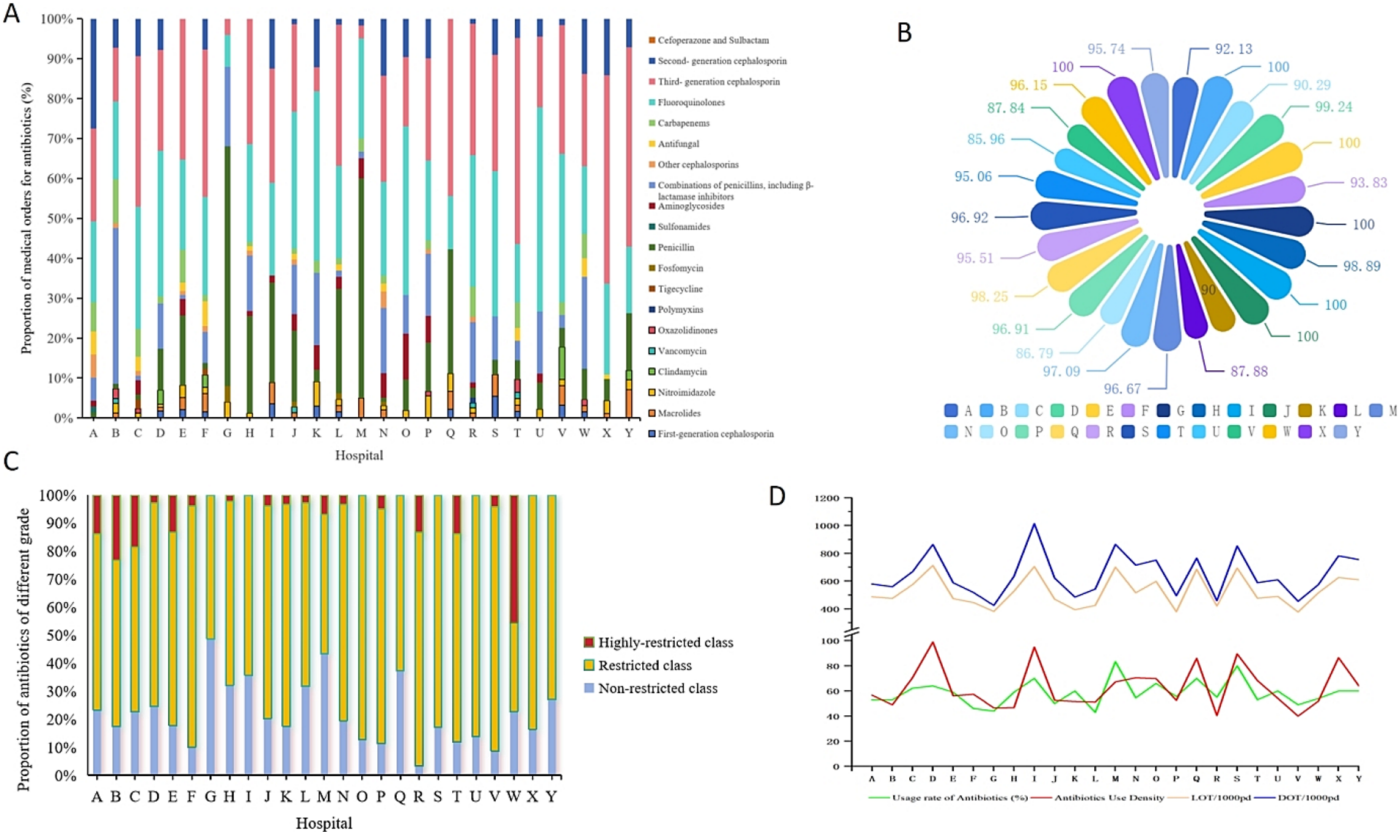
Distribution of antibiotics in each hospital. **(A)** By medical orders; **(B)** by IV administration methods; **(C)** by different restriction level; **(D)** by utilization rate, AUD, LOT and DOT.

Among the 25 hospitals included in this study, the proportion of antimicrobial orders ranged from 6.05 to 21.67% in secondary hospitals and from 7.44 to 19.82% in tertiary hospitals. Figure 1A shows the structural distribution of antimicrobial orders across individual hospitals. Table 1 lists the data on the number of patients, total medication orders, and antimicrobial use. During the study period, the antimicrobial utilization rate exceeded 60% in 7 of the 25 hospitals, with a maximum of 83.33%. In secondary hospitals, this rate ranged from 44.00 to 83.33%, while in tertiary hospitals, it ranged from 43.00 to 64.00%. The antimicrobial utilization rate was generally high across all the hospitals, and significant differences were observed between institutions. Only one hospital met the standard threshold of 40 DDDs/100pd. The AUI in the secondary hospitals ranged from 46.53 to 94.87 DDDs/100pd, while in the tertiary hospitals, it ranged from 40 to 98.99 DDDs/100pd. Statistically significant differences in the AUI were observed among some hospitals (Table 2).

**Table 1:** Percentage of antimicrobial use by hospital, DOT and LOT per 1,000 patient days, and DOT/LOT ratio.

Variable	Total	A	B	C	D	E	F	G	H	I	J	K	L
Number of patients	2064	100	100	98	100	100	100	50	100	50	100	55	100
Usage rate of antibiotics (%)	56.98	53	53	62.25	64	59	46	44	59	70	50	60	43
Antibiotics use density	60.56	56.66	48.92	70.64	98.99	56.11	57.39	46.53	46.7	94.87	52.59	51.60	51.16
Proportion of the number of medical orders for antibiotics (%)	10.66	7.48	7.44	9.54	19.82	11.49	10.52	6.05	10.69	14.43	10.54	10.89	9.14
Proportion of highly restricted antibiotics (%)	3.74	7.14	8.12	9.64	1.29	6.49	2.01	0.00	0.55	0.00	0.44	3.07	0.69
Proportion of items of intravenous infusion medical orders (%)	95.62	92.13	100	90.29	99.24	100	93.83	100	98.89	100	100	90	87.88
Utilization rate of antibiotic intravenous infusion in hospitalized patients (%)	56.97	51.00	53.00	60.20	63.00	59.00	46.00	44.00	58.00	70.00	50.00	56.36	39.00
LOT/1000 pd	508	487	476	575	713	474	446	382	528	705	470	394	425
DOT/1000 pd	622	579	560	668	864	588	518	425	635	1,014	622	485	543
Penicillin	63	20	235	17	79	11	41	81	82	0	73	47	10
Combinations of penicillins, including β-lactamase inhibitors	56	6	1	4	80	69	6	198	163	238	91	3	137
First-generation cephalosporins	7	0	0	0	27	17	20	0	0	41	0	5	5
Second-generation cephalosporin	36	89	46	54	63	0	13	0	0	109	4	23	9
Third-generation cephalosporin	160	117	89	238	148	163	162	14	0	246	110	20	196
Cefoperazone and Sulbactam	83	150	0	119	120	113	104	62	171	63	102	221	9
Other cephalosporins	7	34	5	11	0	3	8	0	9	0	8	0	0
Carbapenems	18	32	53	49	14	52	6	0	9	0	2	11	3
Macrolides	9	0	10	0	2	14	14	0	0	36	10	0	7
Fluoroquinolones	143	95	84	124	310	98	89	41	176	260	172	124	125
Nitroimidazole	9	0	26	5	1	19	8	14	12	0	0	14	2
Sulfonamides	1	11	0	0	0	0	0	0	0	0	0	0	0
Clindamycin	3	0	0	0	18	0	13	0	0	0	0	0	0
Aminoglycosides	15	3	0	14	0	22	0	0	9	22	34	18	28
Vancomycin	1	0	5	0	0	0	0	0	0	0	2	0	0
Linezolid	2	0	6	11	0	0	0	0	0	0	0	0	0
Polymyxins	0	0	0	0	0	0	0	0	0	0	0	0	0
Tigecycline	0	0	0	3	0	0	6	0	0	0	0	0	0
Fosfomycin	1	0	0	0	0	0	0	14	0	0	11	0	4
Antifungal	8	23	0	19	0	6	28	0	4	0	2	0	9
DOT/LOT	1.22	1.19	1.18	1.16	1.21	1.24	1.16	1.11	1.2	1.44	1.32	1.23	1.28

Variable	M	N	O	P	Q	R	S	T	U	V	W	X	Y
Number of patients	54	99	50	99	50	109	50	100	50	100	100	100	50
Usage rate of antibiotics (%)	83.33	54.55	66	55.56	70	55.05	80	53	60	49	54	60	60
Antibiotics use density	67.23	70.53	69.79	52.55	85.99	40.44	89.54	68.29	54.19	40	51.81	86.5	64.01
Proportion of the number of medical orders for antibiotics (%)	13.82	7.89	9.67	15.3	13.87	12.16	21.67	8.37	10.33	8.17	14.36	10.71	11.66
Proportion of highly- restricted antibiotics (%)	2.39	0.81	0.00	2.32	0.00	4.36	0.00	7.09	0.00	1.18	23.92	0.00	0.00
Proportion of items of intravenous infusion medical orders (%)	96.67	97.09	86.79	96.91	98.25	95.51	96.92	95.06	85.96	87.84	96.15	100	95.74
Utilization rate of antibiotic intravenous infusion in hospitalized patients (%)	81.48	54.55	56.00	54.55	70.00	53.21	80.00	51.00	54.00	44.00	54.00	60.00	58.00
LOT/1000 pd	701	516	598	380	686	421	694	477	490	378	515	626	610
DOT/1000 pd	865	715	751	495	766	459	853	589	610	455	574	782	755
Penicillin	8	109	68	88	0	75	43	29	43	6	112	0	0
Combinations of penicillins, including β-lactamase inhibitors	510	4	58	52	181	12	33	13	7	21	40	44	87
First-generation cephalosporin	0	0	0	0	6	0	30	2	0	19	10	0	0
Second-generation cephalosporin	18	66	46	23	0	5	25	12	32	8	64	131	61
Third-generation cephalosporin	31	190	139	113	300	135	223	191	100	139	108	303	368
Cefoperazone and Sulbactam	0	52	8	38	173	39	122	183	144	66	104	70	76
Other cephalosporins	0	41	0	8	0	4	0	0	0	0	0	0	0
Carbapenems	26	13	0	9	0	18	0	32	0	10	24	0	0
Macrolides	52	4	0	0	25	2	30	8	0	21	5	2	45
Fluoroquinolones	182	154	360	99	57	142	347	70	267	135	71	190	97
Nitroimidazole	0	3	10	22	23	5	0	7	7	10	0	24	11
Sulfonamides	0	0	0	0	0	0	0	0	0	0	0	0	0
Clindamycin	0	0	0	0	0	0	0	0	0	21	0	0	11
Aminoglycosides	36	50	64	35	0	16	0	0	11	0	0	0	0
Vancomycin	0	0	0	0	0	4	0	6	0	0	0	0	0
Linezolid	0	0	0	5	0	0	0	20	0	0	3	0	0
Polymyxins	0	0	0	0	0	2	0	0	0	0	0	0	0
Tigecycline	0	0	0	0	0	0	0	0	0	0	0	0	0
Fosfomycin	0	7	0	0	0	0	0	0	0	0	0	0	0
Antifungal	0	21	0	0	0	0	0	16	0	0	32	17	0
DOT/LOT	1.23	1.39	1.26	1.3	1.12	1.09	1.23	1.24	1.25	1.21	1.11	1.25	1.24

**Table 2:** Distribution of 8 types of antimicrobial drug indexes among the three clusters.

Variable	Cluster 1	Cluster 2	Cluster 3	F	*p*
Usage rate of antibiotics (%)	53.01 ± 5.41^a(bc)^	63.65 ± 4.31^b(ac)^	74.33 ± 8.92^c(ab)^	23.65	<0.001
Antibiotics use density	52.84 ± 8.23^a(bc)^	75.39 ± 10.24^b(a)^	87.66 ± 14.16^c(a)^	26.19	<0.001
Proportion of the number of medical orders for antibiotics (%)	10.05 ± 2.52^a(c)^	11.09 ± 1.78^b(c)^	17.44 ± 3.90^c(ab)^	12.52	<0.001
Proportion of highly-restricted antibiotics(%), median (IQR)	2.17[0.59,6.94]	0.00[0.00,4.82]	0.65[0.00,2.12]	-	0.125
Proportion of items of intravenous infusion medical orders (%)	51.35 ± 5.54^a(bc)^	60.84 ± 5.40^b(ac)^	73.62 ± 8.73^c(ab)^	23.07	<0.001
LOT/1000pd	453.69 ± 51.07^a(bc)^	619 ± 41.82^b(a)^	703.25 ± 7.93^c(a)^	-	<0.001
DOT/1000pd, median(IQR)	567[487.50,604.75]a(bc)	755[709.5,774]^b(a)^	864.5[855.75,976.75]c(a)	-	<0.001
DOT/LOT, median(IQR)	1.22[1.17,1.27]	1.24[1.14,1.26]	1.23[1.22,1.39]	-	0.776

^a,b,c^Statistical differences per line (Bonferroni *p*<0.05).

LOT and DOT are quantitative indicators used to monitor antimicrobial use. Among the 25 hospitals included, the LOT of the secondary and tertiary hospitals ranged from 382/1,000pd to 705/1,000pd and from 380/1,000pd to 713/1,000pd, respectively. Statistically significant differences in the LOT were observed across some hospitals (Table 2). The DOT in the secondary hospitals ranged from 425/1,000pd to 1,014/1,000pd, while in the tertiary hospitals it ranged from 455/1,000pd to 864/1,000pd. The top five antimicrobial classes by overall DOT across the 25 hospitals were third-generation cephalosporins (*n* = 24), quinolones (*n* = 25), cefoperazone and sulbactam (*n* = 21), penicillins (*n* = 16), and combinations of penicillins, including *β*-lactamase inhibitors (*n* = 15). The distribution of antimicrobial classes among the top five hospitals included 10 s-generation cephalosporins, 3 macrolides, 5 carbapenems, 3 aminoglycosides, 2 nitroimidazoles, 1 fosfomycin, 1 antifungal, and 1 other cephalosporin.

Among the 1,939 orders for antimicrobial orders for 1,176 patients, all were for systemic agents; no antimicrobials were prescribed. In total, 1,854 orders were for IV administration, while 85 were for oral administration. Figure 1B illustrates the distribution of IV antimicrobial prescriptions across hospitals. Among the 1,176 hospitalized patients using antimicrobials, the overall utilization rate of IV antimicrobial use was 56.97% (1,141/2,064). In the secondary hospitals, the IV antimicrobial utilization rate among inpatients ranged from 44.00 to 83.33%, while in the tertiary hospitals, it ranged from 46.00 to 60.00%. Statistically significant differences in IV antimicrobial utilization were observed among hospitals (Table 2). The route of administration for antimicrobial orders varied greatly among the hospitals, with IV antimicrobials accounting for 100% of orders in four hospitals. Among the 10 types of antimicrobials available in oral formulations, sulfonamides accounted for 100% of their total use. Third-generation cephalosporins had the lowest proportion of oral administration at 0.61%, while linezolid and quinolones—with good oral absorption rates—were administered orally to 28.57 and 8.89%, respectively.

The antimicrobials used in each hospital were grouped according to their clinical application, and the cumulative DDD was calculated. The overall proportion of special-use antimicrobials was 3.74%. A prefecture-level tertiary hospital had the highest proportion at 23.92%. In comparison, the three provincial tertiary hospitals reported proportions of 7.14, 8.12, and 9.64%, respectively. Among the eight hospitals with a 0% usage of special-use antimicrobials, seven were secondary and one was tertiary. Table 1 present the proportion of special-use antimicrobial prescriptions to patients across hospitals. Figure 1C shows the distribution of medical orders by antimicrobial restriction level, indicating a general preference for restricted antimicrobials across all hospitals.

Figure 1D displays the trends in antimicrobial utilization rate, antimicrobial use density (AUD), LOT, and DOT among different hospitals. According to these four indicators, 25 hospitals were ranked, and line charts were drawn. The ranking curve for the AUI of each hospital overlapped with that of LOT/1,000pd. The trend of this curve was slightly different from that of the antimicrobial utilization rate and DOT/1,000pd (Supplementary Figure 1).

**Figure 2:**
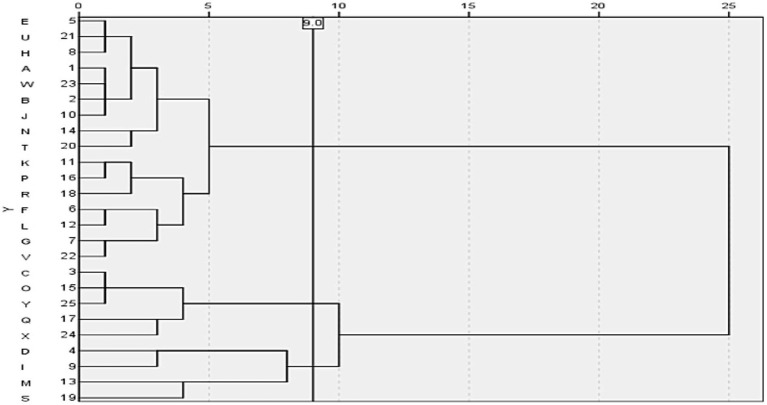
Clustering of the antimicrobial drug indexes. The clustering tree diagram shows the result and effect of clustering analysis; the Y axis reflects the hospitals, and the X axis shows the Semi-Partial R-square (an index in the cluster analysis measuring the heterogeneity). Combining the minimum value of Pseudo T-square (another index in the cluster analysis for selecting best numbers ofclusters), we could cluster these hospitals into three categories.

The AUD serves as the performance assessment metric for public hospitals in China, while DOT and AUD are standardized methods recommended by the World Health Organization for monitoring antimicrobial consumption. The antimicrobial utilization rate and LOT reflect the proportion of individuals administered antimicrobials and their treatment duration, respectively, making them key indicators in antimicrobial monitoring. Therefore, these four indicators were used to perform a systematic cluster analysis of 25 hospitals (Figure 2). When the decision distance was set to 9, hospitals were grouped into three categories. Category 1 included 16 hospitals: A, B, E, H, J, N, T, U, W, F, G, K, L, P, R, and V; Category 2 included 5 hospitals: C, O, Q, X, and Y; and Category 3 included 4 hospitals: D, I, M, and S.

The analysis revealed a disproportionate distribution of antimicrobial use indicators among the 25 surveyed hospitals. A comparative analysis was conducted among the three hospital groups. No statistically significant differences were observed in the proportion of highly restricted antimicrobials (%) and DOT/LOT between groups, while significant differences were observed among other variables. Group 1 exhibited the lowest antimicrobial utilization rate and intensity, as well as the lowest percentage of IV antimicrobial orders (%), IV antimicrobial use among inpatients (%), LOT/1,000pd, and DOT/1,000pd. Conversely, these values were highest in group 3. Group 1 also had the highest proportion of highly restricted antimicrobials (%), whereas group 2 demonstrated the highest DOT/LOT ratio. Table 2 shows the distribution of antimicrobial use indicators across clusters.

### 3.3 Use of antimicrobial agents in different departments

Among the 2,064 patients included in this study, the proportion of antimicrobial orders collected from each department was between 1.80 and 21.60%. The Surgery Department accounted for the largest proportion of antimicrobial orders, followed by the Respiratory Medicine Department, while the Hematology Department had the lowest. Supplementary Tables 1–1, 1–2 show the departmental distribution of antimicrobial orders.

Among all departments analyzed, the Intensive Care Unit (ICU) exhibited the highest antimicrobial utilization rate at 91.67%, followed by the Respiratory Department (87.88%) and the Pediatrics Department (84.55%). Regarding the AUI, the ICU also ranked highest, with 133.28 DDDs/100pd. The Infectious Disease Department followed with an AUI of 120.24 DDDs/100pd., while the Respiratory Medicine, Surgery, and Pediatrics Departments recorded AUIs of 119.00, 105.46, and 53.77 DDDs/100pd, respectively. During the sampling period, AUI in all clinical departments was higher than 40 DDDs/100 pd. The antimicrobial utilization rate and intensity were high across departments, and statistical differences were observed among them.

Supplementary Tables 1-1, 1–2 shows that the Infection Department had the highest antimicrobial exposure across all hospitals; 988 patients were administered antimicrobial therapy per 1,000 hospital days (LOT = 988/1,000pd), followed by the Pediatrics (LOT = 959/1,000pd) and Nephrology (LOT = 962/1,000pd) departments. The Surgery and Respiratory departments reported LOTs of 830/1,000PD and 923/1,000PD, respectively. The ICU showed the highest DOT (1,230/1,000pd), followed by the Infectious Disease (DOT = 1,191/1,000PD) and Respiratory (DOT = 1,163/1,000PD) departments. Supplementary Figure 2 shows the distribution of antimicrobial prescriptions by department. Third-generation cephalosporins ranked among the top five agents by DOT in 12 departments (excluding the ICU). Fluoroquinolones were used in 12 departments but not in Pediatrics. Cefoperazone and sulbactam, penicillins, and penicillin combinations, including *β*-lactamase inhibitors, ranked in the top five in 13, 9, and 8 departments, respectively. Among the top five drugs per department, second-generation cephalosporins appeared in five departments; macrolides appeared in Pediatrics; carbapenems were used in the Nephrology, ICU, and Hematology departments; aminoglycoside in the Infection Department; and antifungal drugs in Hematology.

The IV antimicrobial infusion utilization rate among inpatients ranged from 29.72 to 91.67%. The ICU had the highest utilization rate, while the Neurology department had the lowest. Supplementary Figure 2B displays the distribution of antimicrobial orders through administration routes across different departments. The antimicrobials used in each department were grouped according to their clinical application within their respective hospitals, and cumulative DDDs were calculated. The proportion of special-use antimicrobials in the Hematology department was 37.78%, followed by 18.79% in the ICU. No special-use antimicrobials were prescribed in Pediatrics and Endocrinology. Supplementary Tables 1–1, 1–2 list the proportions of special-use antimicrobials among hospitalized patients in different departments, and Supplementary Figure 2C illustrates the distribution of medical orders via antimicrobial classification for each hospital. Line charts were drawn for DDDs/1,000 pd., LOT/1,000 pd., and DOT/1,000 pd. in each department (Supplementary Figure 2D). The AUI in Pediatrics and LOT in the ICU showed different trends.

## 4 Discussion

Antimicrobial use was investigated among nonsurgical inpatients at 25 medical institutions in Shanxi Province. In total, 2,064 medical records were included. Among these patients, 56.98% were administered at least one antimicrobial during hospitalization—a rate higher than that of the provincial median in 2021 (47.14%) and the range reported in European countries (29–43.9%) (13). The AUI, measured in DDDs, was 60.63, exceeding that of the 2021 provincial average (39.7 DDDs/1,000pd). The cumulative number of DOT for patients was 621/1,000pd, which was also higher than the 576/1,000pd reported in a pre-pandemic study from Brazil. This finding may be related to the large number of patients with COVID-19 admitted during the study period (15). Patients with COVID-19 commonly present with multiple comorbidities and frequent occurrences of lymphocytopenia, which increase the risk of secondary bacterial infections and contribute to poorer clinical outcomes (16, 17). Additionally, the absence of a gold standard for diagnosing or excluding bacterial pneumonia in patients with COVID-19 has led to an increased reliance on empirical antimicrobial therapy in hospitalized populations to a certain extent (18–20). Huang et al. (21) conducted a retrospective case survey during the same period, showing that 83.5% (269/322) of older adults hospitalized patients with COVID-19 were administered empirical antimicrobial therapy. Similarly, international data indicate that 61.8 to 74.6% of hospitalized patients with COVID-19 were treated with antimicrobials. Surgical patients were also excluded from this study, which may account for the high rates and intensity of antimicrobial drugs used by inpatients in this study population.

In 2015, DDD and antimicrobial utilization rates were key control indicators, prompting health authorities at all levels to conduct inspections, evaluations, and assessments of medical institutions. DDD is also used as a performance metric for secondary and tertiary public hospitals to prompt standardized antimicrobial use. Internationally, DOT is a standard monitoring indicator considered equally important as DDD. Both metrics are not affected by individual adjustments, making them suitable for adults, children, and patients with hepatic or renal dysfunction. They are particularly appropriate for pediatric patients whose antimicrobial dosing is based on weight and age. When evaluating antimicrobial use in patients with severe infections, DOT may be the more appropriate measure (22, 23). Although widely used in the United States as a standard measure for antimicrobial use, DOT has some limitations. For example, in patients whose dosing intervals are adjusted based on renal function, the day on which the dosing is skipped is not counted in the total DOT. Therefore, DOT may not adequately represent cumulative antimicrobial exposure, and its quantitative relationship with actual antimicrobial use remains unclear. Further evaluation is needed to determine whether high DOT values reflect a small number of patients undergoing prolonged therapy or a larger number of patients undergoing short treatment courses (24).

In this study, the antimicrobial utilization rate, DDD, DOT, and LOT were used to monitor and compare the overall use of antimicrobials across hospitals and clinical departments. Our results showed that the ranking curve for the DDD closely overlapped with that of LOT/1,000pd, while the curves for antimicrobial utilization rate and DOT/1,000pd showed slight differences. A high inpatient volume during the period may be the primary factor affecting the DDD ranking across all hospitals. The discrepancies between inpatient antimicrobial utilization rates and DOT/1,000pd rankings compared with those of DDDs and LOT/1,000pd suggest that the underlying causes of inappropriate antimicrobial use may vary among hospitals. When interpreted collectively, these four indicators can offer preliminary insights into institution-specific issues in antimicrobial stewardship. For example, all four indicators were ranked high at Hospital G, indicating effective overall control of antimicrobial use. Conversely, Hospital F showed high rankings for antimicrobial utilization rate and DOT but low rankings for DDD and LOT, indicating potential areas for improvement in dosing and treatment duration. At Hospital R, a high antimicrobial utilization rate signals a need for optimization, and further review of medical orders may be warranted. Among clinical departments, pediatrics DD values appeared anomalous, possibly due to the need for weight-based dose adjustments in children (25). In this study, antimicrobial use was evaluated by combining DDD and DOT, each of which offers distinct advantages and disadvantages. Therefore, we advocate the combined use of DDD and DOT as complementary monitoring indicators in antimicrobial drug management to improve the overall prescription quality and optimize antimicrobial drug use.

Analysis of the number of prescriptions, DOT, and DDDs for all categories of antimicrobials across the 25 hospitals revealed that third-generation cephalosporins had the highest number of prescriptions and DDDs, followed by quinolones. This pattern was consistent with trends observed in the CAS data (26). However, the DDD of quinolones during this period was 2.81 times higher than that in the 2022 CAS data (13.6/4.84). The DDD for cephalosporin–*β*-lactamase inhibitor combinations, a key option for the treatment of β-lactamase-producing bacterial infections, increased by 2.9 times (11.54/3.96). This finding aligns with those reported by Nestler et al. (27) during the COVID-19 pandemic. The DDD-based ranking of antimicrobial use was higher than that based on prescription count and DOT, indicating single-dose quantities or use of inappropriate drugs. Moreover, DOT comparisons among hospitals showed statistical differences, indicating the need for targeted case reviews. In this study, carbapenems ranked among the top five antimicrobials in DOT within the ICU and Hematology departments, consistent with national trends reported in the 2023 antimicrobial PPS covering 20 hospitals (28). Additionally, the indicators of the tertiary hospitals were better than those of the secondary hospitals.

In this study, IV antimicrobial therapy was administered to over 85% of patients across different hospitals—a rate significantly higher than the 60.5% reported in Europe (29). The proportion of antimicrobial drug use in each department showed high heterogeneity, ranging from 29.72 to 91.67%. Most physicians opt for IV administration because the patients and clinicians perceive it as more effective, and hospitalized patients are unable to take oral drugs or have contraindications to oral therapy (13, 30). Analysis of the administration routes for specific antimicrobial agents revealed that 8.89, 16.66, and 28.57% of the patients were treated with oral quinolones, oral antifungals, and oral linezolid, respectively. These agents exhibited high oral bioavailability, suggesting that IV use could often be avoided in clinical practice. Oral antimicrobial therapy offers several advantages, including lower infection risk, reduced healthcare costs, and shorter hospital stays (31). Thus, establishing an accurate diagnosis is essential to guide appropriate treatment decisions and ensure the optimal route of administration.

This study has some limitations. Firstly, the analysis was based solely on retrospective case surveys. Secondly, we provided only a descriptive account of antimicrobial use and did not assess the appropriateness of that use. Thirdly, during the COVID-19 pandemic, not all patients with suspected infections underwent microbiological testing due to exceptional circumstances and the strain on hospital systems. Finally, we did not analyze factors such as patient case mix, disease incidence, infection prevalence, or antimicrobial resistance trends, all of which may influence prescribing patterns. Therefore, the findings from this study may not be generalizable to other countries or regions. Nevertheless, this multicenter survey provides useful insights into antimicrobial use within the study region.

In conclusion, we evaluated the utilization rate of antimicrobials, AUD, DOT, and LOT to analyze the antimicrobial use in our province. Antimicrobial utilization was high across hospitals and departments, contributing to increased drug resistance and healthcare costs. A timely response is essential and requires effective communication between clinicians and other healthcare personnel to ensure appropriate diagnosis and treatment, including documentation of prescription stop or review dates and implementation of key interventions, such as stewardship, de-escalation, reducing drug combinations, optimizing the minimum effective daily dose, limiting therapy duration, and preventing nosocomial infections.

## Data Availability

The original contributions presented in the study are included in the article/Supplementary material, further inquiries can be directed to the corresponding authors.

## References

[1] Conly J. Antimicrobial resistance in Canada. CMAJ: Canadian Med Assoc J = journal de l'Association medicale canadienne. (2002) 167:885–91. PMC128402 12406948

[2] Cao W Feng H Ma Y Zhao D Hu X. Long-term trend of antibiotic use at public health care institutions in Northwest China, 2012–20 —— a case study of Gansu Province. BMC Public Health. (2023) 23:27. doi: 10.1186/s12889-022-14944-6, PMID: 36604660 PMC9814306

[3] Domche Ngongang SC Basera W Mendelson M. Tertiary hospitals physician's knowledge and perceptions towards antibiotic use and antibiotic resistance in Cameroon. BMC Infect Dis. (2021) 21:1116. doi: 10.1186/s12879-021-06792-3, PMID: 34715797 PMC8555219

[4] Umeokonkwo CD Oduyebo OO Fadeyi A Versporten A. Point prevalence survey of antimicrobial consumption and resistance: 2015-2018 longitudinal survey results from Nigeria. Afr J Clin Exp Microbiol. (2021) 22:252–9. doi: 10.4314/ajcem.v22i2.18

[5] Da Silva RMR De Mendonça SCB Leão IN. Use of monitoring indicators in hospital management of antimicrobials. BMC Infect Dis. (2021) 21:827. doi: 10.1186/s12879-021-06542-5, PMID: 34404348 PMC8369325

[6] Wang H Wang H Yu X Zhou H Li B Chen G. Impact of antimicrobial stewardship managed by clinical pharmacists on antibiotic use and drug resistance in a Chinese hospital, 2010-2016: a retrospective observational study. BMJ Open. (2019) 9:e026072. doi: 10.1136/bmjopen-2018-026072, PMID: 31377693 PMC6687004

[7] Zheng N Li J Liu Y Liao K Chen J Zhang C. Evaluation of implementation and effectiveness of China's antibiotic stewardship in the first affiliated Hospital of Sun Yat-sen University. Antibiotics (Basel). (2023) 12:770. doi: 10.3390/antibiotics12040770, PMID: 37107132 PMC10135032

[8] Wang L Zhang X Liang X Bloom G. Addressing antimicrobial resistance in China: policy implementation in a complex context. Glob Health. (2016) 12:30. doi: 10.1186/s12992-016-0167-7, PMID: 27267876 PMC4893878

[9] Zhang T Liu C Ren J Wang S Huang X Guo Q. Perceived impacts of the national essential medicines system: a cross-sectional survey of health workers in urban community health services in China. BMJ Open. (2017) 7:e014621. doi: 10.1136/bmjopen-2016-014621, PMID: 28698322 PMC5734402

[10] Wang C Li W Gao J Zhang D Li Y Li F. Microbial predominance and antimicrobial resistance in a tertiary Hospital in Northwest China: a six-year retrospective study of outpatients and patients visiting the emergency department. Canadian J Infectious Dis Med Microbiol = Journal canadien des maladies infectieuses et de la microbiologie medicale. (2020) 2020:1–9. doi: 10.1155/2020/8838447 PMC7719506 33312315

[11] Burrowes SAB Drainoni ML Tjilos M Butler JM Damschroder LJ Goetz MB. Survey of physician and pharmacist steward perceptions of their antibiotic stewardship programs. Antimicrobial Stewardship & Healthcare Epidemiol: Ashe. (2021) 1:e48. doi: 10.1017/ash.2021.219, PMID: 36168491 PMC9495632

[12] CiŽman M Plankar ST. Antibiotic consumption and resistance of gram-negative pathogens (collateral damage). GMS Infectious Dis. (2018) 6:Doc05. doi: 10.3205/id000040 PMC6301726 30671336

[13] Xie DS Xiang LL Li R Hu Q Luo QQ Xiong W. A multicenter point-prevalence survey of antibiotic use in 13 Chinese hospitals. J Infect Public Health. (2015) 8:55–61. doi: 10.1016/j.jiph.2014.07.001 25129448

[14] Rawson TM Moore LSP Zhu N Ranganathan N Skolimowska K Gilchrist M. Bacterial and fungal coinfection in individuals with coronavirus: a rapid review to support Covid-19 antimicrobial prescribing. Clin Infect Dis. (2020) 71:2459–68. doi: 10.1093/cid/ciaa530, PMID: 32358954 PMC7197596

[15] Antunes BBP Silva AAB Nunes PHC Martin-Loeches I Kurtz P Hamacher S. Antimicrobial consumption and drug utilization patterns among Covid-19 and non-Covid-19 patients. J Antimicrob Chemother. (2023) 78:840–9. doi: 10.1093/jac/dkad025, PMID: 36740939

[16] Lai CC Chen SY Ko WC Hsueh PR. Increased antimicrobial resistance during the Covid-19 pandemic. Int J Antimicrob Agents. (2021) 57:106324. doi: 10.1016/j.ijantimicag.2021.106324, PMID: 33746045 PMC7972869

[17] Grau S Hernández S Echeverría-Esnal D Almendral A Ferrer R Limón E. Antimicrobial consumption among 66 acute care hospitals in Catalonia: impact of the Covid-19 pandemic. Antibiotics (Basel). (2021) 10:943. doi: 10.3390/antibiotics10080943, PMID: 34438993 PMC8388964

[18] Ghosh S Bornman C Zafer MM. Antimicrobial resistance threats in the emerging Covid-19 pandemic: where do we stand? J Infect Public Health. (2021) 14:555–60. doi: 10.1016/j.jiph.2021.02.011, PMID: 33848884 PMC7934675

[19] Mustafa ZU Saleem MS Ikram MN Salman M Butt SA Khan S. Co-infections and antimicrobial use among hospitalized Covid-19 patients in Punjab, Pakistan: findings from a multicenter, point prevalence survey. Pathogens Global Health. (2022) 116:421–7. doi: 10.1080/20477724.2021.1999716, PMID: 34783630 PMC9518253

[20] Zhou C Jiang Y Sun L Li H Liu X Huang L. Secondary pulmonary infection and co-infection in elderly Covid-19 patients during the pandemics in a tertiary general hospital in Beijing. China Front Microbiol. (2023) 14:1280026. doi: 10.3389/fmicb.2023.1280026, PMID: 37901822 PMC10600495

[21] Saleem Z Haseeb A Godman B Batool N Altaf U Ahsan U. Point prevalence survey of antimicrobial use during the Covid-19 pandemic among different hospitals in Pakistan: findings and implications. Antibiotics (Basel). (2022) 12:70. doi: 10.3390/antibiotics12010070, PMID: 36671271 PMC9854885

[22] Ababneh MA Jaber M Rababa'H A. Prevalence of antimicrobial use in a tertiary academic hospital: a venue for antimicrobial stewardship programs. Expert Rev Anti-Infect Ther. (2021) 19:1047–51. doi: 10.1080/14787210.2021.1863789, PMID: 33307895

[23] Kallen MC Natsch S Opmeer BC Hulscher MEJL Schouten JA Prins JM. How to measure quantitative antibiotic use in order to support antimicrobial stewardship in acute care hospitals: a retrospective observational study. European J Clin Microbiol Infectious Dis: Official Pub European Society Clin Microbiol. (2019) 38:347–55. doi: 10.1007/s10096-018-3434-0, PMID: 30478815

[24] Vallès J Fernández S Cortés E Morón A Fondevilla E Oliva JC. Comparison of the defined daily dose and days of treatment methods for evaluating the consumption of antibiotics and antifungals in the intensive care unit. Med Int. (2020) 44:294–300. doi: 10.1016/j.medin.2019.06.008 31378384

[25] Montecatine-Alonso E Mejías-Trueba M Goycochea-Valdivia WA Chavarri-Gil E Fernández-Llamazares CM Dolz E. Development of antimicrobial defined daily dose (Ddd) for the pediatric population. Antibiotics (Basel). (2023) 12:276. doi: 10.3390/antibiotics12020276, PMID: 36830187 PMC9952639

[26] Wushouer H Zhou Y Zhang W Hu L du K Yang Y. Inpatient antibacterial use trends and patterns, China, 2013-2021. Bull World Health Organ. (2023) 101:248–261B. doi: 10.2471/BLT.22.288862, PMID: 37008266 PMC10042087

[27] Nestler MJ Godbout E Lee K Kim J Noda AJ Taylor P. Impact of Covid-19 on pneumonia-focused antibiotic use at an academic medical center. Infect Control Hosp Epidemiol. (2021) 42:915–6. doi: 10.1017/ice.2020.362, PMID: 32698920 PMC7590557

[28] Xiao Y Xin X Chen Y Yan Q The China PPS team. A comprehensive point prevalence survey of the quality and quantity of antimicrobial use in Chinese general hospitals and clinical specialties. Antimicrob Resist Infect Control. (2023) 12:127. doi: 10.1186/s13756-023-01334-9, PMID: 37974231 PMC10652455

[29] Al Matar M Enani M Binsaleh G. Point prevalence survey of antibiotic use in 26 Saudi hospitals in 2016. J Infect Public Health. (2019) 12:77–82. doi: 10.1016/j.jiph.2018.09.003, PMID: 30270148

[30] Umeokonkwo CD Madubueze UC Onah CK Okedo-Alex IN Adeke AS Versporten A. Point prevalence survey of antimicrobial prescription in a tertiary hospital in south East Nigeria: a call for improved antibiotic stewardship. J Global Antimicrobial Resistance. (2019) 17:291–5. doi: 10.1016/j.jgar.2019.01.013, PMID: 30668994

[31] Versporten A Zarb P Caniaux I Gros MF Drapier N Miller M. Antimicrobial consumption and resistance in adult hospital inpatients in 53 countries: results of an internet-based global point prevalence survey. Lancet Glob Health. (2018) 6:e619–29. doi: 10.1016/S2214-109X(18)30186-4, PMID: 29681513

