# Supplementary material for: Use of multiple metrics and clustering analysis to assess antimicrobial use in Shanxi hospitals, China: a cross-sectional study based on 25 general hospitals
Source: Front Public Health. 2025 Aug 13;13:1464613. doi: 10.3389/fpubh.2025.1464613 (PMC12380702; doi:10.3389/fpubh.2025.1464613)
Supplement: Supplementary file 1 [file Supplementary_file_1.docx]

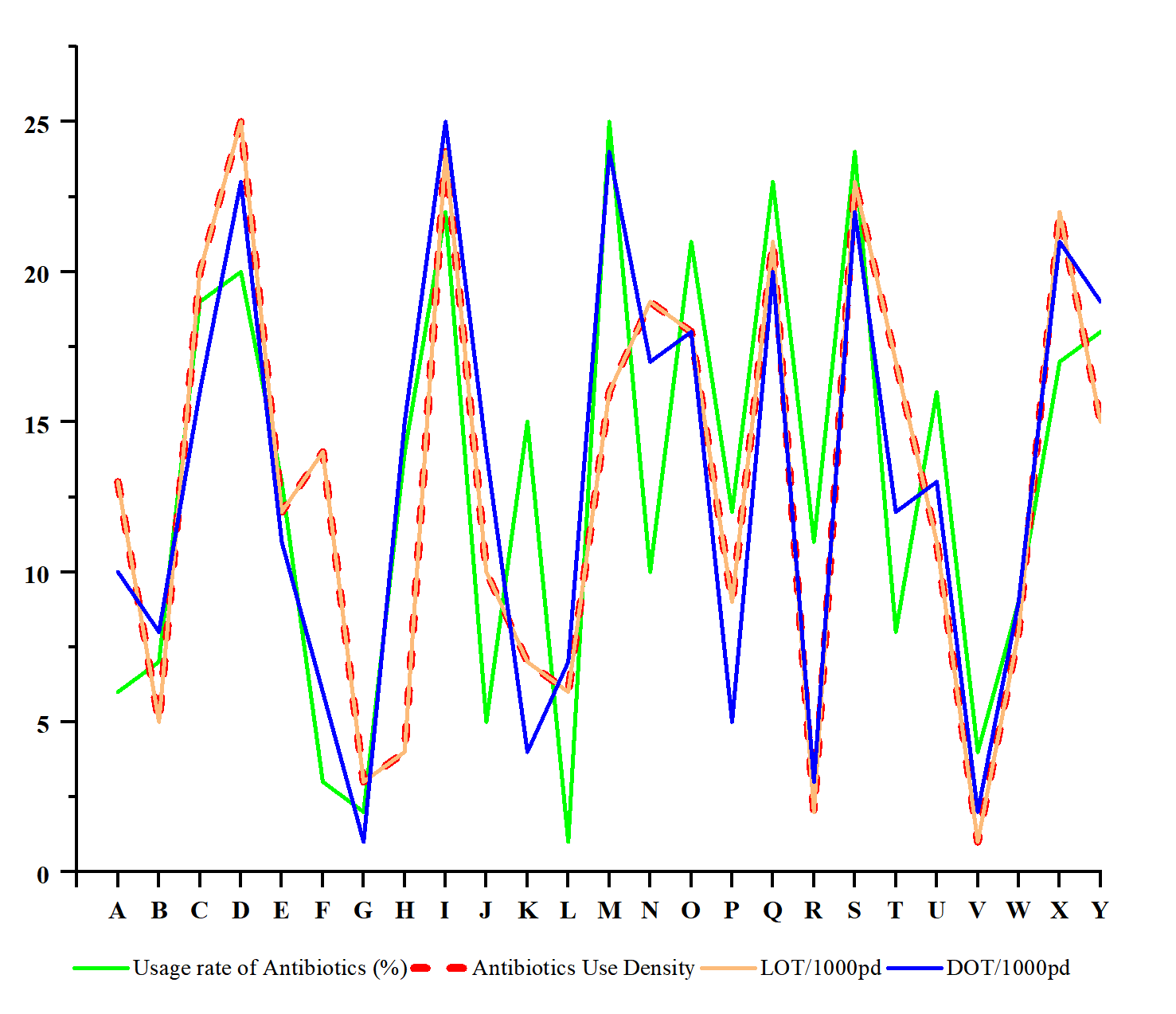


**Supplemental Figure 1.** Line chart of the ranking of the four indicators for each hospital


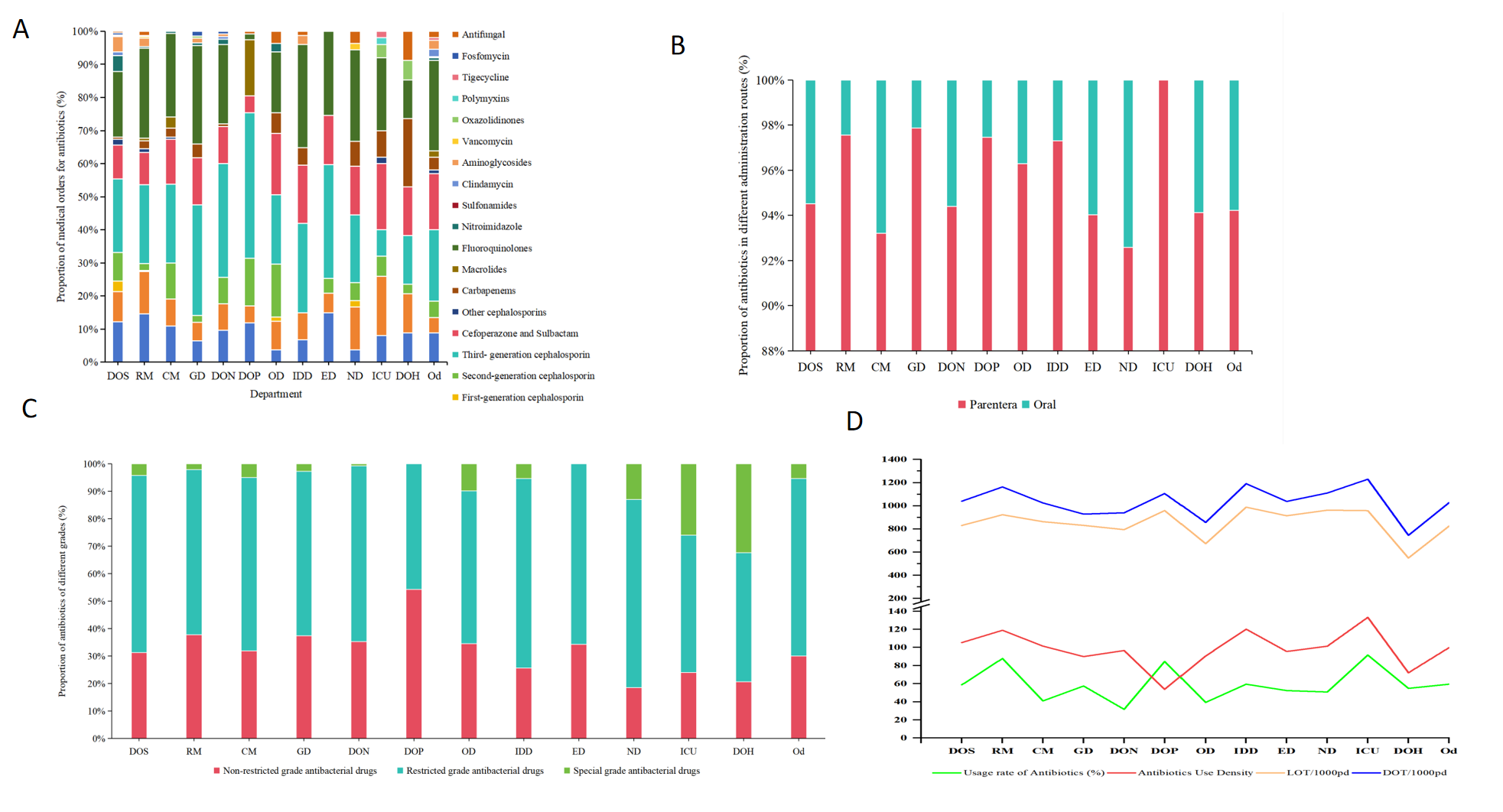


**Supplemental Figure 2** Distribution of antibiotics in each department. (A) by medical orders; (B) by IV administration methods; (C) by different restriction level; (D) by utilization rate, AUD, LOT and DOT. Department of surgery:DOS,Respiratory medicine:RM,Cardiovascular medicine:CM, Gastroenterology department:GD,Department of neurology:DON,Department of pediatrics:DOP,Oncology department:OD,Infectious disease department:IDD,Endocrinology department:ED,Nephrology department:ND,Intensive care unit:ICU,Department of hematology:DOH,Other departments:Od

**Supplemental Table1-1.** Percentage of antimicrobial use by department, DOT and LOT per 1000 patient days, and DOT/LOT

| Variable | DOS | RM | CM | GD | DON | DOP |
| --- | --- | --- | --- | --- | --- | --- |
| Number of patients | 451 | 231 | 222 | 160 | 249 | 110 |
| Usage rate of Antibiotics (%) | 58.76 | 87.88 | 40.99 | 57.5 | 31.73 | 84.55 |
| Antibiotics Use Density | 105.46 | 119 | 101.54 | 89.96 | 96.64 | 53.77 |
| Proportion of the number of medical orders for antibiotics（%） | 21.6 | 19 | 7.6 | 7.3 | 6.4 | 6.1 |
| Proportion of highly- restricted antibiotics（%） | 1.59 | 2.91 | 1.06 | 2.61 | 1.05 | 0.00 |
| Proportion of items of intravenous infusion medical orders（%） | 94.51 | 97.56 | 93.2 | 97.87 | 94.4 | 97.46 |
| Utilization rate of antibiotic intravenous infusion in hospitalized patients（%） | 56.10 | 87.01 | 39.64 | 57.50 | 29.72 | 84.55 |
| LOT/1000pd | 830 | 923 | 863 | 831 | 794 | 959 |
| DOT/1000pd | 1040 | 1163 | 1025 | 928 | 940 | 1106 |
| Penicillin | 113 | 162 | 95 | 59 | 122 | 138 |
| Combinations of penicillins, including β-lactamase inhibitors | 108 | 161 | 75 | 73 | 82 | 48 |
| First-generation cephalosporin | 45 | 1 | 0 | 0 | 0 | 0 |
| Second-generation cephalosporin | 66 | 22 | 114 | 12 | 77 | 131 |
| Third-generation cephalosporin | 226 | 282 | 307 | 341 | 294 | 488 |
| Cefoperazone and Sulbactam | 130 | 113 | 92 | 136 | 108 | 75 |
| Other cephalosporins | 26 | 12 | 4 | 0 | 0 | 0 |
| Carbapenems | 5 | 34 | 20 | 32 | 10 | 0 |
| Macrolides | 0 | 3 | 45 | 0 | 0 | 174 |
| Fluoroquinolones | 196 | 313 | 269 | 243 | 218 | 0 |
| Nitroimidazole | 53 | 9 | 4 | 7 | 8 | 0 |
| Sulfonamides | 0 | 0 | 0 | 0 | 0 | 0 |
| Clindamycin | 8 | 0 | 0 | 0 | 6 | 0 |
| Aminoglycosides | 48 | 35 | 0 | 11 | 8 | 0 |
| Vancomycin | 2 | 4 | 0 | 0 | 0 | 0 |
| Linezolid | 3 | 0 | 0 | 4 | 0 | 0 |
| Polymyxins | 0 | 0 | 0 | 0 | 0 | 0 |
| Tigecycline | 0 | 0 | 0 | 0 | 0 | 0 |
| Fosfomycin | 9 | 0 | 0 | 0 | 7 | 0 |
| Antifungal | 2 | 13 | 0 | 10 | 0 | 29 |
| DOT/LOT | 1.25 | 1.26 | 1.19 | 1.12 | 1.18 | 1.15 |

**Supplemental Table1-2.** Percentage of antimicrobial use by department, DOT and LOT per 1000 patient days, and DOT/LOT (continued table)

| Variable | OD | IDD | ED | ND | ICU | DOH | Od |
| --- | --- | --- | --- | --- | --- | --- | --- |
| Number of patients | 117 | 74 | 84 | 57 | 24 | 31 | 254 |
| Usage rate of Antibiotics (%) | 39.32 | 59.46 | 52.38 | 50.88 | 91.67 | 54.84 | 59.45 |
| Antibiotics Use Density | 90.59 | 120.24 | 95.63 | 101.44 | 133.28 | 72.06 | 99.78 |
| Proportion of the number of medical orders for antibiotics（%） | 4.2 | 3.8 | 3.5 | 2.8 | 2.6 | 1.8 | 13.4 |
| Proportion of highly- restricted antibiotics（%） | 8.25 | 2.85 | 0.00 | 8.04 | 18.79 | 37.78 | 3.37 |
| Proportion of items of intravenous infusion medical orders（%） | 96.3 | 97.3 | 94.03 | 92.59 | 100 | 94.12 | 94.23 |
| Utilization rate of antibiotic intravenous infusion in hospitalized patients（%） | 38.46 | 56.76 | 50.00 | 49.12 | 91.67 | 54.84 | 57.87 |
| LOT/1000pd | 673 | 988 | 914 | 962 | 958 | 549 | 824 |
| DOT/1000pd | 857 | 1191 | 1038 | 1111 | 1230 | 745 | 1026 |
| Penicillin | 7 | 96 | 140 | 57 | 57 | 50 | 86 |
| Combinations of penicillins, including β-lactamase inhibitors | 70 | 79 | 45 | 143 | 191 | 59 | 40 |
| First-generation cephalosporin | 13 | 0 | 0 | 30 | 0 | 0 | 0 |
| Second-generation cephalosporin | 142 | 0 | 32 | 63 | 183 | 9 | 46 |
| Third-generation cephalosporin | 152 | 304 | 407 | 239 | 42 | 95 | 224 |
| Cefoperazone and Sulbactam | 154 | 204 | 151 | 182 | 260 | 77l | 213 |
| Other cephalosporins | 0 | 0 | 0 | 0 | 42 | 0 | 19 |
| Carbapenems | 52 | 46 | 0 | 81 | 73 | 187 | 33 |
| Macrolides | 0 | 0 | 0 | 0 | 0 | 0 | 18 |
| Fluoroquinolones | 203 | 362 | 263 | 263 | 321 | 131 | 257 |
| Nitroimidazole | 22 | 0 | 0 | 0 | 0 | 0 | 6 |
| Sulfonamides | 0 | 0 | 0 | 0 | 0 | 0 | 7 |
| Clindamycin | 0 | 0 | 0 | 0 | 0 | 0 | 18 |
| Aminoglycosides | 0 | 83 | 0 | 0 | 0 | 0 | 31 |
| Vancomycin | 0 | 0 | 0 | 15 | 0 | 0 | 0 |
| Linezolid | 0 | 0 | 0 | 0 | 31 | 71 | 0 |
| Polymyxins | 0 | 0 | 0 | 0 | 11 | 0 | 0 |
| Tigecycline | 0 | 0 | 0 | 0 | 19 | 0 | 2 |
| Fosfomycin | 0 | 0 | 0 | 0 | 0 | 0 | 0 |
| Antifungal | 42 | 17 | 0 | 39 | 0 | 65 | 26 |
| DOT/LOT | 1.27 | 1.2 | 1.14 | 1.16 | 1.33 | 1.36 | 1.24 |

Department of surgery:DOS,Respiratory medicine:RM,Cardiovascular medicine:CM, Gastroenterology department:GD,Department of neurology:DON,Department of pediatrics:DOP,Oncology department:OD,Infectious disease department:IDD,Endocrinology department:ED,Nephrology department:ND,Intensive care unit:ICU,Department of hematology:DOH,Other departments:Od
